# An Exploratory Study of Research Needed for the Improvement of Care and Services for Persons with a Lived Experience of Mental Health Challenges

**DOI:** 10.3390/ijerph23030342

**Published:** 2026-03-09

**Authors:** Anton Isaacs, Sharon Lawn, Anna Baker

**Affiliations:** 1 School of Rural Health, Monash University, Warragul, VIC 3820, Australia; 2 College of Medicine and Public Health, Flinders University, Adelaide, SA 5001, Australia; sharon.lawn@flinders.edu.au; 3 Lived Experience Australia, Adelaide, SA 5048, Australia; 4 Mental Health Services, Latrobe Regional Hospital, Traralgon, VIC 3844, Australia

**Keywords:** lived experience research, health services needs and demand, mental health services, health services research, healthcare quality, access and evaluation, caregivers, patients, service users

## Abstract

**Highlights:**

**Public health relevance—How does this work relate to a public health issue?**

- This research relates to care and services for individuals with severe mental health challenges.
- Despite decades of mental health policy and practice reform activity, as well as mental health research, which have together involved significant public health resources, there has been limited evidence of improved service quality for people with mental health challenges.

**Public health significance—Why is this work of significance to public health?**

- For people with mental health challenges, and for their careers and families, issues of equity, disempowerment, tokenism, and exclusion from decision-making and involvement in shaping public health policy persist.
- This work highlights that lived experience perspectives are necessary in determining what is researched, and co-design must occur to achieve effective public health outcomes for this population.

**Public health implications—What are the key implications or messages for practitioners, policy makers and/or researchers in public health?**

- The lived experience perspective demonstrates that optimal care for individuals with severe mental health challenges extends beyond current mental health service delivery and includes a system-wide approach.
- Leadership of persons with lived experience in research sheds light on how systems could better support and be held accountable to those with a lived experience of mental health challenges.

**Abstract:**

Individuals with a lived experience are increasingly being included in the design of mental health services research. Obtaining perspectives of persons with a lived experience of mental health challenges on research that is important to them is an opportunity to achieve equity in allocating resources so that policy-makers and health research funders are made aware of the issues that matter to people who are affected by research. The purpose of this study is to explore lived experience perspectives on research needed for the improvement of care and services for persons with mental health challenges in order to improve the quality of care and service delivery. This qualitative study was conducted in Australia and was informed by biographical research and interpretive phenomenological analysis [IPA]. Twenty-one participants were interviewed for the study. The theme, ‘Access to care and early detection’ included eight categories. The theme, ‘Care and treatment’, included fifteen categories. The theme, Continuity of care’, included six categories. Research questions were developed for each category and serve as a first step towards initiating research on the identified topics. Research topics identified in this study were categorized as insufficiently researched, emerging areas of interest, or well researched with insufficient translation into practice.

## 1. Introduction

A person’s mental health is shaped by various social, economic, and physical environments that operate at different stages of life. Improving and maintaining one’s mental health therefore requires action to improve the conditions of everyday life [1]. Recent literature has highlighted that people experiencing severe mental health challenges [SMHCs] have several unmet needs that are not directly related to mental health services but have an impact on their personal recovery [2]. These needs, however, are frequently neglected or misunderstood within a healthcare system that is predominantly structured for episodic care [3]. Mental health services [MHSs] research seeks to address these unmet needs and enhance service quality. To design care and services that are truly person-centered, it is imperative that persons with a lived experience of mental illness are actively engaged in their development. (In this paper, we use the terms ‘persons with lived experience’ [PWLE], ‘individual’, and ‘client’ interchangeably to refer to individuals who experience mental health challenges [4]). However, research in mental health and mental health services remains heavily influenced by a ‘reductionist biomedical model,’ which continues to dominate clinical practice, policy, research agendas, and investment in mental health globally [5]. This research aims to amplify the voices of PWLE [4] and to illustrate that mental health services research must also be approached from the perspective of lived experience.

Australia’s mental health system encompasses a diverse array of clinical and non-clinical services [6]. Public MHSs include area-based clinical MHSs, which comprise both inpatient and community MHSs. In addition, there are mental health community support services that are non-clinical services provided by non-governmental organizations, as well as private MHSs (psychiatrists and psychologists) and the National Disability Insurance Scheme (NDIS). Mental Health Community Support Services also operate adult prevention and recovery care (PARC) services, which are community-based, short-term supported residential services for people experiencing mental health problems but do not need acute care. These services are staffed by visiting mental health nurses and doctors.

The primary stakeholders in the design and implementation of contemporary mental health services (MHSs) have traditionally been policymakers and mental health professionals within both practice and academia [7]. While mental health professionals have been reported to be particularly concerned with ‘risk,’ specifically the risk of violence, policymakers are primarily focused on the distribution of resources [7]. PWLEs have emerged as a third influential group, seeking to shape the delivery of MHSs based on their ‘expertise by experience’ [7]. Various important reports have shown that current MHSs have several shortcomings including challenges in accessibility, a tendency to ‘operate in crisis’ with insufficient emphasis on personal recovery, fragmented services that fail to support caregivers and families, inadequate funding, neglect, and issues of affordability [8,9].

Despite the implementation of Australia’s 1992 National Mental Health Strategy, which focused on deinstitutionalization, community mental health, better accountability and the delivery of human rights for people with a mental illness and carers [10], there has been limited evidence of improved service quality [11]. Currently, accreditation and policy guidelines require services to follow standard protocols, with professionals needing to complete significant amounts of paperwork, thereby limiting their time to meaningfully engage with PWLEs. Conversely, individuals express frustration with challenges that include the insufficient involvement of family members in their care and the emphasis of services on reaching a diagnosis rather than addressing the various factors that significantly impact on their condition and contribute to their persistence [12].

### 1.1. PWLE Involvement in MHSs Research

It has been argued that mental health service design and implementation is at the threshold of a paradigm shift that is similar to that of deinstitutionalization [13], with the voices of PWLEs and carers being given equal importance in their design. As an extension, PWLEs have also been involved in MHSs research. PWLE involvement in MHSs research has in large part been due to the contributions of the recovery movement, where individuals with a psychiatric diagnosis are treated as persons with hopes and aspirations, dignity, and autonomy rather than as cases who have no capacity to understand their illness [14]. Meaningfully involving individuals in research that impacts them not only benefits them by enabling empowerment and facilitating recovery but also improves the quality of research in terms of its validity and usefulness [14].

The level of involvement of PWLEs in MHSs research has been gradually increasing. Initially, studies focused on individuals’ experiences with mental health care [15]. More recently, methods of co-design and co-production of research have been documented [16], and such co-produced research has been shown to enhance individuals’ experiences of mental health care [17]. PWLE leadership in research has provided insights into how systems could better support and be accountable to those with a lived experience of mental health challenges [18]. These leadership roles also offer opportunities to leverage perspectives and experiences derived from lived experiences [18]. However, despite these advancements, issues of tokenism and power imbalances persist in research conducted in collaboration with PWLEs [19].

### 1.2. A Conceptual Model

Tansella and Thornicroft offer a conceptual model (matrix model) to guide the process of MHSs reform [20]. The model has two dimensions—a geographical dimension that includes country, local, and individual levels, and a temporal dimension that includes input, process, and outcome phases [20], all of which intersect with each other. Their research focuses on the individual level at the input, process, and outcome phases. However, Tansella and Thornicroft describe this level mostly from the perspective of the psychiatrist or mental health clinician. It is possible that there are aspects of care that are problematic for PWLEs, but which psychiatrists and other mental health clinicians do not recognize because they are part of a system built through education and organizational protocols that mostly view mental health care through a clinical lens.

### 1.3. Rationale for the Study

There is a growing realization of a commonly occurring mismatch between research questions that are important to investigators and those that are relevant to PWLEs [21].

There are also ongoing demands for reform of Australian mental health services that is informed by lived experience. In its draft statement on consumer and community involvement in health and medical research, the National Health and Medical Research Council [NHMRC] of Australia has stated that the lived experiences of individuals and their perspectives must be respected [22]. This study aims to unpack the perspectives of PWLE by following their biography or illness journey. Experiences of their illness journey related to MHSs that are important to individuals are not easily forgotten, and topics for future research and subsequent areas for future improvement can be ascertained from these experiences.

## 2. Materials and Methods

### 2.1. Co-Design

This study was co-designed and conducted with PWLE. A co-design approach refers to the involvement of PWLE in the design of products or services to better serve their intended purpose [23]. This research was led by a senior mental health services researcher and co-designed with two individuals who have a lived experience of mental health challenges. Co-investigator SL is a lived experience research professor whose work focuses on systems of mental health care, the culture of service provision, implementation of services, and individual experiences of care. SL is also the Chair of Lived Experience Australia. Co-investigator AB is a lived experience worker at a regional mental health service and has co-authored previous research in this field. The concept for this work emerged during discussions between the lead author and co-author SL. While SL provided guidance on the methodology, both co-investigators assisted the lead author in data analysis to ensure that participant responses were understood and represented as accurately as possible. In addition, draft results were reviewed by a separate lived experience advisory group. The details of this process and its outcomes are described later.

### 2.2. Study Design

This was a qualitative study informed by biographical research [24] and interpretive phenomenological analysis [IPA] [25]. Biographical research aims to classify and explain social processes through biographies [24]. This methodology utilizes life history interviews as the primary data collection instrument [26]. Interviewees are asked to tell their story with no other specific instructions. These interviews are therefore designed to generate ‘a spontaneous autobiographical narration which is not structured by questions posed by the interviewer but by the narrator’s structures of relevance’ [27]. While the life history is being narrated, the interviewer does not interrupt. Instead, they use non-verbal and para-linguistic expressions to demonstrate interest and attention [27]. Only after the narrator completes their story does the interviewer ask questions comprising the second part of the interview [27]. These questions are based on reconstructions of the critical junctures in their life course. The answers to these questions reflected participants’ lived experience and their contexts. Analysis of these lived experience accounts was informed by IPA, where the researcher interpreted the meanings and context of the participant’s experience to formulate research questions [25].

### 2.3. Participants and Data Collection

Determining a sample size for this study was challenging since it was clear that participant experiences would be numerous and varied as there are people with mental health challenges. Therefore, setting a sample size to include all types of experiences was not practical. Furthermore, the focus of this study was not to develop a theory but to highlight the reality that topics for research also needed to be informed by individual needs and experiences.

Participants were recruited by Lived Experience Australia (a voluntary organization that advocates for better MHSs) from their national ‘friends’ network of several hundred members who are PWLEs or carers or both. An advertisement to participate was included in their newsletters and social media. Those who were interested in participating in the study contacted the first author, who provided them with the explanatory statement and consent forms. Since this study was advertised in a newsletter subscribed by hundreds of PWLEs and carers, it was only possible to identify those who responded to the call. No participant dropped out after submitting their signed consent form. Mutually convenient interview times and dates were decided on receipt of signed consent forms.

Once the interviewer (lead author) established rapport with general conversation and confirmed their ability to participate in the interview, participants were asked to tell their story from the time they perceived that they began to experience distress and develop signs of mental health problems. Throughout the narrative, the interviewer noted down, a posteriori, critical junctures in their story where care and services were either missing or could have been improved. This required an understanding of individual needs and MHS design. Once the narration was complete, the interviewer drew their attention to each critical juncture identified during the narration and asked them three questions given below:

At that point in time,

1. what, if anything, do you think you would have liked to know?
2. what if anything would you have liked others to know?
3. what opportunities if any, would you have liked for you to be able to make your own decisions (personal agency)?

All three questions were asked one after another, giving the participant the opportunity to answer as many as they felt comfortable or able to. For example, once a participant finished narrating their story, the interviewer drew attention to a critical juncture in their experience where they said, ‘I spent weeks telling the psychologist my story but no help…’, and asked them to reflect on the questions above. The participant responded by stating, ‘Psychologists need to be trauma-informed. They do not necessarily understand suffering and poverty…’. Once all key experiences were addressed, participants were asked if they had anything additional to convey. Any responses were recorded. Interviews lasted between 60 and 90 min. Interviews were audio-recorded. Participants received AUD 40 in compensation for their time.

### 2.4. Data Analysis

Although the entire interview was audio-recorded. Only participant’s responses to the questions were transcribed and analyzed for this study. These transcripts were anonymized and treated as follows. Informed by IPA, the first author labeled participant responses as an ‘experience’ or ‘message’ to be conveyed or both. Responses were labeled as an ‘experience’ when it related to the participant’s experience. Responses were labeled as a ‘message’, when it was directed outward and related to how necessary services were missing or existing services could be improved. These experiences and messages were subjected to inductive thematic analysis. They were initially coded by the first author. The two co-authors with a lived experience (SL and AB) independently reviewed and modified the coded data as needed.

Codes were inductively combined to form categories. Once all data were coded, they were deductively classified into three pre-determined themes: (1) Access to care and early detection, (2) Care and treatment, and (3) Continuity of care. These themes were aligned to individual experiences with MHSs that typically fall under the broad areas of access to care [28], the actual delivery of health care and treatment [29], and continuity of care [30] rather than clinical perspectives and presentations [31]. From the perspective of the individual, delivery of care is experienced as care and treatment. Once thematic analysis was complete, each investigator then contributed research questions to each category. In this way, the phenomenon being studied was the participants ‘message’ as a response to their experience. The researcher’s interpretation of the ‘message’ took the form of proposing relevant research questions for each message. In keeping with the co-design process, we did not impose any further analytical structures that could take away the essence of what participants were saying.

Draft results were then presented for review to a separate lived experience advisory group consisting of six individuals from the national lived experience representative panel of Lived Experience Australia. Of the six group members who reviewed the results, four returned their comments and suggestions by email. A group meeting was conducted to discuss the results. Advisory group members made some revisions to the existing questions as well as added further questions. Contributions from advisory group members were denoted as AG-number (e.g., AG-3). Advisory group members received an honorarium of AUD 60 for their time (reading and meeting). The authors assert that all procedures contributing to this work comply with the ethical standards of the relevant national and institutional committees on human experimentation and with the Helsinki Declaration of 1975, as revised in 2013.

## 3. Results

Twenty-one participants were interviewed for the study. The majority of participants (n = 14, 67%) were female and aged between 40 and 49 (n = 9, 43%). Nine participants (43%) had a graduate qualification and were involved in an occupation related to their lived experience of mental health challenges. Seventeen (81%) participants did not have a carer. Ten (48%) were hospitalized for their mental illness at least once and thirteen (62%) had experienced mental health challenges for more than 20 years. Further details are given in Table 1. Members of the lived experience advisory group had lived experience as individuals, family carers, or both and were involved in systemic advocacy.

A brief description of the three themes of Access to care and early detection, Care and treatment, and Continuity of care is given below. Research questions that were generated from the experiences and messages of participants are given in Table 2, Table 3 and Table 4. Quotes of experiences and messages within each category are given in the Supplementary Tables S1–S3.

### 3.1. Access to Care and Early Detection

The theme, ‘Access to care and early detection,’ encompasses eight categories and pertains to barriers in seeking assistance, missed opportunities for early identification, and inadequately designed services. Research questions and the categories they relate to within this theme are listed in Table 2. Within this theme, participant experiences and narratives indicate that stigma continues to be a substantial obstacle to accessing mental health services (MHSs). Participants also identified factors contributing to mental health challenges in adulthood, such as childhood sexual abuse and the absence of screening and detection of mental health issues during school years. The process of seeking assistance during a mental health crisis emerged as a critical topic, involving both police procedures during crisis assessment and treatment, as well as the emergency department’s (ED) procedures when engaging with individuals in crisis. While the former caused considerable distress to individuals, the latter were described as insensitive and inappropriate. Participants noted that mental health issues often impacted families through vicarious trauma, stress, career disruptions, and consequent financial challenges, while family MHSs are scarce. Similarly, long waiting times for service access and the strategies employed by individuals to overcome these barriers were identified as areas warranting further research. Topics related to family caregivers also emerged, including the lack of information on the impacts of becoming a caregiver and the insufficient support available once they assumed this role. Supplementary Table S1 provides quotes related to experiences and messages for each category under this theme.

**Table 2:** Research questions for the theme: Access to care and treatment.

**Stigma and Discrimination**
How is stigma experienced in everyday contact with services, and what are its impacts? How does the experience of shame interact (or not) with the experience of self-stigma for persons with mental health challenges? How would you prefer to have been responded to when reaching out?—AG2 How is shame experienced in everyday contact with services, and what are its impacts? What contributes to shame for people with mental health issues? Do persons with mental health problems feel shame due to not having their true needs met, or heard by providers? How can researchers best explore issues of stigma and shame with people with lived experience of mental health concerns? How can stigma associated with mental health services be reduced?
**Child sexual abuse [CSA] and adverse childhood events**
What are the experiences of persons when disclosing CSA?—AG2 How can we create ways for individuals and healthcare providers to feel safe and helpful about raising and talking about CSA? What do individuals want healthcare providers to say or ask them about past trauma or abuse? How can child abuse be identified early in children? What are the experiences of seeking justice for CSA for people with mental health concerns?
**School mental health**
How can positive cultures of mental health support be developed among school students?/How can mental health and wellbeing be a normalized topic in schools? Is there an association between tolerance and diversity in schools and mental health and wellbeing of students? How are family inclusive culture and practices implemented in schools? What is the impact of schoolteachers as mentors on resilience building for young people in the school context? Does awareness training in child mental health for parents improve early diagnosis and treatment? -AG1 What is the association between leadership styles of school leaders and teachers and the culture of school communities? How effective are school mental health programs? How can school counseling and pastoral care services be enhanced to include early identification of mental health challenges from a lived experience perspective? What does mental health peer support look like in the school yard?
**Crisis assessment and treatment**
What are better and safer ways of helping individuals who become unwell with mental health problems at home? What are safe alternatives to Crisis Assessment and Treatment Teams in regional areas? (Specific to the Australian context and may be relevant for other settings) What are the short and long-term impacts of coercive treatment and contact when people are in crisis? What training do police and emergency services get in regard to responding to a person in mental health crisis? How does it align with training received by mental health care providers facing the same in a clinical setting? What aspects of crisis care do people in distress value the most and least? What aspects of crisis care do families/carers of people in distress value the most and least? What are the different models of crisis assessment and treatment by multi-disciplinary teams? How effective are they in improving patient centered outcomes?—AG1 What roles do peer support workers play in crisis support?
**Emergency Department [ED] and mental health care**
What are the attitudes of mental health staff at EDs towards people with mental health problems? How can emergency care for people who are suicidal be improved? How can EDs and staff be more sensitive to exchanges of information to ensure privacy and confidentiality? What are the safe space/Safe Haven models and how effective are they?—AG3 What can EDs learn from Safe Havens? How can Triage in the ED be improved?
**Lack of services for families**
How can community models such as the ‘village’ approach to support be built to support mental health and connection in the community? How can mental health services be enhanced to include care of the family? What factors contribute to family invisibility in the Triangle of Care? How can family involvement in care be better validated and formalized? How effective are family mental health care models?
**Waiting lists**
What strategies do individuals employ to get entry into mental health services? How does a “No wrong door” approach work?
**Information and psychosocial support for carers**
What information should a person have when they become a carer for an individual with severe mental illness? What psychosocial supports do carers need outside of mental health services? How does becoming a mental health carer impact identity within and beyond the carer role? What distinguishes mental health caring from other forms of caring for another person? What are the implications? (for the person, the carer, services, communities) What are the experiences of vicarious trauma and Post-Traumatic Stress Disorder among careers?—AG3 What further supports are required for carers (e.g., housing and Centrelink)—AG3 (Specific to the Australian context and may be relevant in other settings) What does the Carer journey look like? What if any are the stages? What impact does it have on their wellbeing and future? How are transitions managed?—AG3

### 3.2. Care and Treatment

The theme, ‘Care and treatment’, included fifteen categories that related to quality of care delivered by the different elements of the mental health system. Research questions related to each of the categories are listed in Table 3. In this theme, participants highlighted the pronounced disparities in the quality of care between private and public MHSs and emphasized the necessity for some degree of resemblance and comparability. They also discussed the challenges in locating clinicians, particularly psychologists, with whom they felt understood, safe, and confident to work with. This was deemed essential due to the considerable variation in approach and focus among psychologists in private practice, resulting in individuals often needing to consult multiple practitioners before finding one with whom they felt comfortable.

Participants further reported on certain deficiencies in the structure of psychological services and the factors that hindered positive outcomes. Some expressed their reluctance to rely on medication and the distress experienced when staff insisted on such treatments rather than exploring alternatives. Participants also recounted experiences and conveyed messages related to treatment in psychiatric wards, including instances of inhumane treatment by staff. They suggested that simple adjustments, such as providing an orientation to the ward layout and routine, could significantly enhance the inpatient experience.

Additionally, they noted that healthcare providers frequently lacked awareness of the impacts of trauma on their clients and the methods to provide care for those who had endured it, underscoring the need for enhanced training on the subject. The necessity for assistance from a psychosocial advocate during a mental health crisis was expressed by participants to help individuals avoid making detrimental decisions while maintaining their personal agency. They also underscored the importance of incorporating peer support workers into the care provided by MHSs.

The lack of service integration and the need for care coordination were other topics highlighted by participants, who also noted the perceived lack of knowledge regarding mental health issues and services among general practitioners, psychologists, emergency services, and the police. Supplementary Table S2 provides quotes of experiences and messages for each category under this theme.

**Table 3:** Research questions for the theme: Care and Treatment.

**Private vs. Public System**
What makes a clinic/hospital environment more or less able to support persons with mental health problems when they are unwell? What are the differences between public and private inpatient services for mental health, from the user’s perspective? How can the differences be reduced? What are the key improvements required for the public mental health system to provide better care? What are the guidelines followed when booking appointments at bulk billing services? How can they be improved for existing clients?
**Feeling safe with healthcare providers**
What steps can mental health services and mental health professionals take to ensure that their clients feel safe and confident to share their story? How do mental health professionals distinguish between what is important and what is not, from their client’s presenting problems? What are the differences between the understanding of a therapeutic relationship between clients and therapists?—AG2 How are agency, autonomy and power navigated by people with mental health concerns and what are the consequences? How does one choose the right psychologist without first having to try them out? What factors promote a connection between individuals and their psychologists/mental health professionals? What therapeutic practices, modalities and personal qualities of healthcare providers are the best fit for PWLEs?—AG3 Why do individuals need to feel a connection with their psychologists/mental health professionals? Trust—how is it built, exchanged, lost for people with mental health concerns when seeking help from health professionals? What does evidence for effectiveness of therapy look like from a lived experience perspective? How can choice and control be better given to the individual?
**Improving private psychological services**
What makes psychological therapy useful? How can private psychology services be made more outcome oriented? What do PWLEs expect from their therapist? What are the barriers to therapy that are experienced by psychologists?—AG3 How many sessions are needed with the Psychologist for therapy to be useful? How can psychological therapy standards be implemented? What supports do individuals say are needed between psychology appointments?—AG2 Would a wider tele health service assist in creating a greater choice of services in the community?—AG1
**Client choice in type of medication**
What are the barriers experienced by people with mental health problems in popping pills? What alternatives exist to popping pills for people with mental health challenges? What role does choice in treatments play for people with mental health challenges? How do they experience the interface with doctors when discussing medication concerns? How do people self-manage and adapt in their daily lives in relation to offered medications and other treatment options? What is the evidence on the effectiveness of shared decision-making in medication prescriptions?—AG3 How often is shared decision-making practiced? What are the barriers to its implementation?—AG3 What are the barriers to implementing social prescribing? What parts of treatment do individuals get a say in? Does this differ from flexibility in treatment choice allowed in individuals wanting treatment for a physical illness?
**ED and mental health care**
What are the elements of care needed for persons in a mental health crisis? What are the service characteristics necessary to care for an individual experiencing a mental health crisis? What are the best ways of implementing the Peer-First, Peer-Last models of care? How, if ever, do they vary depending on type of mental health setting? What are the non-ED models of care (e.g., Psychiatric Emergency Care centers and Safe Havens) for individuals in a mental health crisis? How effective are they? How can care for persons in a mental health crisis be improved? What lessons can the public system learn from the non-governmental sector on emergency mental care?
**Interactions with staff in the psychiatry ward**
What do mental health clients feel about the way that mental health staff interact with them? What do clients feel about how mental health staff should interact with them? How, if ever, can individuals who are anxious be made to feel safe in psychiatric wards? What outcomes do mental health interventions aim to achieve in their clients? What outcomes should mental health interventions aim to achieve in their clients? What roles can allied health professionals play in the care of persons with mental health challenges? How is gender-based discrimination experienced by individuals when they access mental health services? How are gendered assumptions operationalized in mental health care? How can socioeconomically disadvantaged clients be better helped? What factors prevent healthcare professionals from sharing health related information with clients? How do people with lived experience define and understand the concept of health literacy? What are the views of staff and patients regarding the culture of the ward environment (milieu)? Why do people with mental health problems get blamed by services? Does training of mental health staff reduce blaming? What do people need to build confidence in long-term self-care of their mental health? How can people with lived experience and mental health professionals effectively share power and expertise? What are the workforce challenges faced by mental health services?—AG1
**Improving inpatient care**
How can care in inpatient psychiatry wards be improved? How can mental health wards be humanized? What is the patient milieu in the inpatient ward? What are the subcultures of recovery? What are the hidden cultures of patient-to-patient peer support while admitted to psychiatry wards? What do staff think patients are doing during their time in inpatient units? How are inpatient units and social worlds navigated? Do the boundaries required for staff at mental health wards impact the patient experience and their recovery process?—AG2 How does burnout in mental health staff impact patient recovery?—AG2 What mental health supports are available for mental health workers?—AG2 Many mental health nurses I talked to got into the field due to a mental health diagnosis in themselves or others close to them. Does the work retraumatize them? How can we support them so they can support consumers?—AG2
**Trauma-informed care**
How do service cultures and practices inform the delivery of trauma-informed care? What does mental health trauma look like in everyday exchanges with healthcare providers? What is and is not appropriate language to use with individuals who experience mental health problems? What training do mental health professionals receive in ‘trauma-informed care’? How do they put this training into practice? Would buddying up GPs with mental health peer workers enhance the former’s understanding of mental health challenges?—AG1 What is the best way to teach doctors about trauma and trauma-informed-care for people who experience mental health challenges? What gets in the way of healthcare providers understanding the presence of trauma and the potential impacts of their practice? What are the elements of trauma-informed care? How can people with mental health conditions assert themselves safely within mental health systems? from the perspectives of mental health providers, consumers and family/carers? Do they align? Why or why not? What do every day human rights look like in the ED for people with mental health conditions?
**Need for a psychosocial advocate during a mental health crisis**
What psychosocial supports are needed for people during times of crisis? How could individuals be better supported by allied health professionals when experiencing a mental health crisis?—AG1 What would advanced care plans look like from a lived experience perspective? How effective are advanced care plans from a lived experience perspective?—AG3
**Peer involvement**
What if any, is the role of mental health peer work in GP and psychologist practices? What roles do mental health peer workers play in mental health services? How does the mental health peer work role differ in different mental health settings and contexts? What are the common elements of peer support across mental health settings and contexts? What qualifications or professional development would mental health peer workers benefit from, to enhance their skillset to ensure a duty of care is met within clinical mental health settings? AG1
**More integration of care**
How can the different sectors of the mental health service system work together? How can patient notes/records be shared between providers of mental health care, treatment and support?
**Career acknowledgement and support**
How must mental health services engage with carers of individuals with mental health problems? Why are carers not valued and believed by mental health professionals and services? What are the psychosocial support needs of carers of persons with severe mental health challenges? What barriers are carers experiencing when attempting to engage or engaging with ‘Carers Gateway’ and ‘Carers Australia’ to access psychosocial supports?—AG1 (Specific to the Australian context and may be relevant in other settings) How can carer Strategies, Policies and Charters be realized in practice?
**Training for health professionals**
What can people with lived experience teach doctors about mental health care? How do doctors include lived experience into the decisions they make about treatment and care? What are GP’s views of their knowledge of mental health problems? What are the views and experiences of International GPs on treatment of mental disorders? What are GP’s attitudes towards mental health presentations, mental health care and training in mental health?—AG1 How effective is trauma-informed care training for psychologists? What, if any training, do the police receive in mental health care?
**Child protection agencies and mental health care**
What, if any mental health training does child protection service staff receive? Is there a need to improve training? What are the experiences of new mothers with midwifery services when they have mental health problems? Would engaging mental health peer workers with child protection workers enhance outcomes?—AG1
**Hope**
How do people with mental health conditions define hope? How can hope be instilled in people with lived experience of severe and enduring mental health problems? Would engaging with allied health professionals with lived experience enhance hope in consumers?—AG1

### 3.3. Continuity of Care

The theme, Continuity of care’, included six categories that related to care following discharge and interactions with other agencies. The related research questions are listed in Table 4. Within this theme, participants recounted experiences and conveyed messages regarding the unmet need for ongoing support following the discharge of individuals from hospitals. This included the necessity for enhanced mental health support for men, the needs of caregivers transitioning from full-time employment to a full-time caregiving role, and their subsequent transition back to regular life. Participants also detailed their interactions with Centrelink staff (Centrelink is the Australian social welfare agency), the challenges associated with securing housing, and the support provided by the NDIS for individuals with SMHCs. Supplementary Table S3 provides quotes of experiences and messages for each category under this theme.

**Table 4:** Research questions for the theme: Continuity of care.

**Post-Discharge Care and Support**
What supports are needed for individuals who are discharged from the psychiatric hospital from the client perspective? What are care transition needs from a lived experience perspective? Would gender specific care enhance outcomes post admission?—AG1 What supports are needed for new mothers who are discharged from the psychiatric hospital? What type of care do maternal and child nurses provide for mothers with mental health problems?
**Men’s mental health service needs**
What are men’s mental health support needs? Is mental health recovery experienced differently for men, compared with women?
**Career needs and support**
What policies do employers have that relate to when their employees experience mental health challenges or become carers of individuals with mental health challenges? How are these policies put into practice? How can carers regain an identity beyond their caring role after several years of caring? How do carers develop a new identity once their caring role ends? How do they navigate this change? Would more training for employers around mental health assist carers or employees transition to and from the workforce?—AG1
**Centrelink interactions**
How does Centrelink engage with individuals with mental health problems? (Specific to the Australian context and may be relevant in other settings) What should the assessment process for Disability Support Pension look like from a lived experience perspective? What would make it better for the person and family carers? (Specific to the Australian context and may be relevant in other settings) What, if any training does Centrelink employees receive on engaging with persons experiencing mental health challenges? (Specific to the Australian context and may be relevant in other settings) What is the role of a Centrelink social worker? AG1 (Specific to the Australian context and may be relevant in other settings)
**Housing**
What factors influence housing for persons with mental health challenges? What do people with mental health challenges say improves their housing tenure? What barriers do mental health consumers experience when accessing accommodation services?—AG1
**NDIS supports**
How does NDIS support individuals with psychosocial disability from a client perspective? (Specific to the Australian context) What mental health training does NDIS support workers need to have? (Specific to the Australian context) What are the benefits and challenges of receiving support from non-mental health trained support workers from a client perspective? What are the roles of the NDIS support coordinator? What are the competencies they need—AG1 (Specific to the Australian context)

## 4. Discussion

This study sought to explore lived experience perspectives on research topics for the improvement of care and services for persons with SMHCs. Previous studies have utilized other methods such as World Café [32] and Leximancer analysis [33]. This study was different in that it asked questions that were specific to the critical junctures identified in the participants’ illness journey, whereas previous papers asked more general questions of their participants, such as “ What sort of research would you like to see prioritized in a national research agenda?” [32] or “share three things mental health research should focus on” [34]. The results of this study draw attention to research topics that fall into three categories. (1) Topics that might not have been considered previously, (2) topics that have already been identified but where research is still emerging, and (3) topics that have attracted extensive research, the findings of which, have not been widely translated into practice. The findings add to the emerging literature on lived experience perspectives on research to improve care and services for people with SMHCs.

### 4.1. New Research Topics

There are different types of psychological services and a wide variation in how psychological services are delivered. There are also some resources to help individuals find a psychologist to suit their needs [35]. However, unlike seeking help from GPs for physical ailments, choosing the right psychologist can be challenging because ‘People are not going to tell their story unless they feel safe and confident.’ Furthermore, the impacts of ‘retelling the story’, the financial consequences of obtaining new referrals and paying new initial consultancy fees, as well as the potential to use up their ten subsidized sessions on their mental health care plan with little therapeutic progress needs exploration. There are calls for more widespread adoption of measurement-based care (MBC), which involves the evaluation of individual symptoms before and during psychological therapy in order to monitor change [36]. The compelling recognition of personal recovery as a goal of treatment by MHSs means that PWLE-reported outcome measures focusing on personal recovery need to be introduced as well [37].

New research is also needed for topics such as the impacts of interactions with staff in the psychiatry ward; the need for psychosocial advocacy during the time of mental health crisis; the role of child protection agencies in maternal mental health care; post-discharge care and support; and individual interactions with Centrelink. While the topic of waiting lists is more related to demand and supply, the various tactics individuals use to gain entry to an almost inaccessible public mental health service, might be worth exploring. Similarly, research could focus on improving public MHSs using lessons learnt from the private sector.

### 4.2. Emerging Research Topics

While the long-term adverse mental health outcomes of child sexual abuse and adverse childhood events have been documented [38], further research is needed to improve early detection strategies and devise methods to mitigate medium- to long-term mental health impacts. Mental health crisis assessment and treatment was another common topic identified by participants. Although research in this area is emerging [39], the focus of the research is mostly clinical in nature and does not consider lived experience perspectives. As such, research into more nuanced crisis assessment and treatment procedures is needed. Mental health care in the emergency department has also attracted research. For example, alternatives to the ED for persons in a mental health crisis have been proposed [40].

Improving care in psychiatry wards is also an emerging topic of research. The ‘Safewards’ model first developed in the UK provides direction for research in this area, although studies report that PWLE perspectives on the model are not yet fully understood [41]. Another emerging area of research is men’s mental health [42] where further studies are required on men’s mental health service needs. Other emerging research topics include family mental health, client choice in medication type, mental health training programs for non-mental health professionals and housing for persons with mental health problems.

### 4.3. Well-Researched Topics

Topics identified by participants that have already been extensively researched include mental health stigma and discrimination related to mental health and mental health care [43]. However, implementing behaviour change to reduce stigma continues to be a distant dream. Similarly, carer needs and support have attracted extensive research. Despite the existence of best practice guidelines on carer engagement [44] and voluntary organizations dedicated to carer needs [45], MHSs have yet to fully appreciate the value of carers in the care of individuals with SMHCs. School mental health is another widely researched topic [46], although on-the-ground services appear to be inadequate. The need for lived experience workforce within MHSs is widely acknowledged with the publication of National Lived Experience Workforce Guidelines [47]. Nonetheless, there continue to be barriers to the acceptance of their role and the professionalism that should be accorded to it [48]. Integration of MHSs has been a topic of research for several decades and barriers to integration have been reported previously [49]. However, overcoming barriers to integration requires systems-level change.

The crucial gap in knowledge that this study has sought to fill is the inclusion of the voice of PWLE, in what is otherwise a topic dominated by clinician perspectives. Tansella and Thornicroft’s matrix model for mental health reform starts with entry to services, while the LE perspective starts with challenges faced in early recognition of mental health challenges in the community, stigma attached to mental illness, and barriers to service access, all of which take place long before the stage of service entry. Similarly, the focus on the process of care is enhanced to include the previously overlooked disparities that exist between the quality of care between the private and the public system, the way individuals are treated in services, choice and control in treatment, and the important role of carers and peers in care. Finally, while outcomes are typically measured on discharge from the mental health service, lived experience perspectives highlight the importance of continuing care and support that requires the involvement of non-medical support agencies that must also provide support to carers.

An important finding from this study is that topics that are important to persons with SMHCs exceed far beyond the scope of MHSs as we know them, thereby underscoring the need for a broader, more holistic approach to care that includes community inclusion and connection. The impacts of SMHCs appear to be less well understood or perhaps attract less attention than those of severe and chronic physical challenges. This study therefore upholds the need for a paradigm shift in approach from “What’s the matter with you?” to “What matters to you” [50]. The findings of this study also reiterate the need for a person-centered approach where all relevant services are aware and trained to provide appropriate care. Finally, this study has re-emphasized the importance of a public mental health strategy that promotes positive mental health and wellbeing, addresses challenges such as stigmatization of mental health problems, and renews focus on families, schools, and workplaces [51].

The questions generated from this study are crucial for identifying and subsequently addressing existing gaps in implementation science that stem from the neglect of lived experience perspectives during the initial stages of research design. This oversight significantly affects the successful translation of research outcomes into practical applications, as well as the efficient utilization of limited resources to ensure maximum impact on PWLEs and carers.

Furthermore, these questions may help to inform local and national research strategies by highlighting, from a lived experience perspective, key issues relevant to mental health service planning, provision, and evaluation that are important to individuals, families, and communities. The topics identified could serve as a foundational starting point for more detailed consultations and co-design efforts with relevant stakeholders. These would include considerations of their applicability to local contexts, needs, and priorities that require attention.

This study has some limitations. The research questions generated from participant experiences and messages are by no means comprehensive, but rather a sample to provide direction for future research. Participants were mostly middle-aged females who were connected to advocacy networks. Indigenous, culturally, and linguistically diverse and other disengaged groups were missed. Including these groups would have required specifically targeted recruitment strategies. There were also fewer male participants. Men’s mental health service needs might be very different from that of females. The predominance of qualitative questions is due to topics being mostly exploratory but is also a reflection of the expertise of the research team. Readers are encouraged to develop their own questions based on their interest, expertise, type of setting, and service needs of the population they work with. Although further analysis of themes and experiences would have enriched the findings, this was avoided for the sake of brevity. Future research in this field may need to explore views of disadvantaged communities in different settings followed by consensus endeavors such as Delphi studies.

## 5. Conclusions

This study explored lived experience perspectives of research needed for the improvement of care and services for PWLEs. Research questions were developed for three main themes: Access to care and early detection, Care and treatment, and Continuity of care. Topics identified in this study were either insufficiently researched, emergent or were well researched with insufficient translation into practice. The findings of this study draw attention to topics that were specifically of importance to PWLEs and included those that were beyond the scope of MHSs. The results demonstrate that optimal care for persons with SMHCs requires a system-wide approach. Proposed research questions aimed to set the foundation on which further research can be designed based on local needs and expertise.

## Figures and Tables

**Table 1:** Demographic characteristics of participants (n = 21).

Age in Years	Frequency	Presence of Career	Frequency
30–39	1	Yes	4
40–49	9	No	17
50–59	6	Diagnosis *	
60–69	4	Major Depressive Disorder	15
70+	1	Post-traumatic Stress Disorder	12
**Gender**		Anxiety Disorder	8
Male	7	Schizoaffective Disorder	1
Female	14	Borderline Personality Disorder	2
**Highest education**		Attention Deficit Hyperactive Disorder	4
High School	1	Complicated grief	1
Certificate	2	Autism Spectrum Disorder	2
Diploma	4	Obsessive–Compulsive Disorder	2
Graduate	9	Bipolar Affective Disorder	3
Postgraduate	5	Alcohol Addiction	1
**Occupation**		Panic Disorder	1
No Occupation	3	**Duration of illness (in years)**	
Librarian	1	0–9	2
Recovery Support Worker (NDIS)	1	10–19	6
Program coordinator	1	20–29	7
Lived experience consultant	4	30–39	4
Consumer advocate	2	More than 40	2
Peer Support Worker	3	**Number of hospitalizations**	
Volunteer	1	0	11
Accountant	1	1–4	6
Marketing	1	5–9	3
Lawyer	1	More than 10	1
Social Worker	1		
General Manager	1		

* Each diagnosis has been listed separately since most participants had multiple diagnoses.

## Data Availability

The data presented in this study are available on request from the corresponding author due to privacy and confidentiality issues.

## Supplementary Materials

The following supporting information can be downloaded at https://www.mdpi.com/article/10.3390/ijerph23030342/s1. Table S1: Categories and research questions for the theme: Access to care and early detection; Table S2: Categories and research questions for the theme: Care and Treatment; Table S3: Categories and research questions for the theme: Continuity of care.
