# Supplementary material for: An Exploratory Study of Research Needed for the Improvement of Care and Services for Persons with a Lived Experience of Mental Health Challenges
Source: Int J Environ Res Public Health. 2026 Mar 9;23(3):342. doi: 10.3390/ijerph23030342 (PMC13026143; doi:10.3390/ijerph23030342)
Supplement: Supplementary file 1 [file ijerph-23-00342-s001.zip › ijerph-4121839-supplementary.pdf]

Supplementary Tables

Table S1: Categories and research questions for the theme: Access to care and early detection

Experience/ Message	Possible research questions
**Stigma and discrimination**	
Experience - I did not seek help until it got out of hand because there is so much of stigma and shame. – P1M<br>*Message* - Mental health is also health – just different – P1M	How is stigma experienced in everyday contact with services, and what are its impacts?<br>How does the experience of shame interact (or not) with the experience of self-stigma for persons with mental health challenges? How would you prefer to have been responded to when reaching out? - AG2<br>How is shame experienced in everyday contact with services, and what are its impacts?<br>What contributes to shame for people with mental health issues?<br>Do persons with mental health problems feel shame due to not having their true needs met, or heard by providers?<br>How can researchers best explore issues of stigma and shame with people with lived experience of mental health concerns?<br>How can stigma associated with mental health services be reduced?
**Child sexual abuse [CSA] and adverse childhood events**	
Experience - I was abused as a child by my father. My mother put a padlock on my door but my father broke the door down. I wasn't able to tell anyone about my abuse. – P19F<br>*Experience* - I was abused so many times by my uncle and I was too young to understand what was going on. I was just a very shy girl.<br>*Message* - There must be a routine screening of children for trauma in primary schools. Perhaps if it is picked up early…– P18F<br>*Experience* - I reported my child abuse to the police when I was 35 but the experience was very stressful. I was admitted several times to the hospital during the court case. – P19F<br>*Message* - Professional approach it's about trauma informed approach – we don't ask questions they tell us – it's about creating a safe environment. I hear many disclosures in my role - AG1	What are the experiences of persons when disclosing CSA? - AG2<br>How can we create ways for individuals and healthcare providers to feel safe and helpful about raising and talking about CSA?<br>What do service users want healthcare providers to say or ask them about past trauma or abuse?<br>How can child abuse be identified early in children?<br>What are the experiences of seeking justice for CSA for people with mental health concerns?
**School mental health**	
Experience - I didn't have any friends in school… I didn't know about my diagnosis.	

I just didn't have energy to complete assignments– P8M<br>*Message* - School mental health needs to be developed – P8M<br>*Experience* - Nobody picked up on my anxiety… So I dismissed my mental health problems… pushed it to the back of my mind. – P5F<br>*Experience* - I started drinking at 14 to manage my depression and anxiety – P17F<br>*Message* - I wish there were adults at school who could recognise my mental health problems and have assessed and treated them. – P17F<br>*Message* - I kept being sent to the sick bay in primary school. There was no language to describe my symptoms-P7F<br>*Message* – There are lots of discussions in class around mental health... It could be far better funded by the govt --- kids are waiting weeks to see a psych or social worker…… external providers may be a choice. Also awareness training for parents… - AG1<br>*Experience* - I started taking medication only at age 32. – P8M<br>*Message* - Mental health problems need to be identified early– P8M	How can positive cultures of mental health support be developed among school students?/ How can mental health and wellbeing be a normalised topic in schools?<br>Is there an association between tolerance and diversity in schools and mental health and wellbeing of students?<br>How are family inclusive culture and practices implemented in schools?<br>What is the impact of school teachers as mentors on resilience building for young people in the school context?<br>Does awareness training in child mental health for parents improve early diagnosis and treatment? - AG1<br>What is the association between leadership styles of school leaders and teachers and the culture of school communities?<br>How effective are school mental health programs?<br>How can school counselling and pastoral care services be enhanced to include early identification of mental health challenges from a lived experience perspective?<br>What does mental health peer support look like in the school yard?
**Crisis assessment and treatment**	
Experience - When the mental health service was called because I became unwell, the police arrived; handcuffed me, kicked me in my stomach and dragged me out of the house like a criminal in front of the neighbours…– P1M<br>*Message* - I will never advise anyone with a mental health problem to inform the mental health service when they are unwell. Regional areas have no CAT teams…– P1M<br>*Experience* - When I got unwell at home, the police came and took me to hospital in handcuffs… I was sent home the next day-P7F	What are better and safer ways of helping individuals who become unwell with mental health problems at home?<br>What are safe alternatives to Crisis Assessment and Treatment Teams in regional areas?<br>What are the short and long-term impacts of coercive treatment and contact when people are in crisis?<br>What training do police and emergency services get in regard to responding to a person in mental health crisis? How does it align with training received by mental health care providers facing the same in a clinical setting?<br>What aspects of crisis care do people in distress value the most and least?<br>What aspects of crisis care do families/carers of people in distress value the most and least?
Message - I didn't want anyone turning up to my house making a scene. You know how it is, when people come to do welfare checks… Welfare checks need to be done discreetly. – P10F	What are the different models of crisis assessment and treatment by multi-disciplinary teams? How effective are they in improving patient centered outcomes? – AG1

	What roles do peer support workers play in crisis support?
**Emergency Department [ED] and mental health care**	
Experience - I hated going to the Emergency department when I was unwell and had to speak loudly to a nurse on the other side of the glass telling them that I was feeling suicidal…. Everyone else staring at you. – P1M<br>*Message* - ED is not an appropriate place for persons who are mentally unwell. – P1M<br>*Message* - ED is not appropriate for someone with mental health. *– P5F*<br>*Message* - Offer the alternative to request a triage room. Most EDs have a separate room. - AG1	What are the attitudes of mental health staff at EDs towards people with mental health problems?<br>How can emergency care for people who are suicidal be improved?<br>How can EDs and staff be more sensitive to exchanges of information to ensure privacy and confidentiality?<br>What are the safe space/ Safe Haven models and how effective are they? - AG3<br>What can EDs learn from Safe Havens?<br>How can Triage in the ED be improved?
**Lack of services for families**	
Experience - There is no help for families– P9M<br>*Message* - We need a village model. There are many people in the community who have resources and knowledge and experience to offer support. – P9M<br>*Experience* - Families are not included in care and are informed only when the patient is ready for discharge– P5F<br>*Message* - A lot of the challenges need to be addressed by the family– P5F<br>*Message* - Include family members/carers in the process offering peer support to family members. It's tricky with privacy laws - AG1	How can community models such as the 'village' approach to support be built to support mental health and connection in the community?<br><br>How can mental health services be enhanced to include care of the family?<br>What factors contribute to family invisibility in the Triangle of Care? How can family involvement in care be better validated and formalised?<br>How effective are family mental health care models?
**Waiting lists**	
Experience - Waiting lists are a major barrier to access. Once has to act up to get into services– P4F<br>*Message* - It is difficult to get admission unless you say that you are suicidal – P15M	What strategies do service users employ to get entry into mental health services?<br>How does a "No wrong door" approach work?
**Information and psychosocial support for carers**	
Experience - When I suddenly became a carer, nobody told me about the devastating impacts it would have on me emotionally, mentally, financially, relationally. My marriage broke down, I was left with little money. I was a wreck. – P3F<br><br>*Message* - We need information on what can happen when someone goes from living a regular life to suddenly becoming a carer. It should be like a cancer diagnosis. – P3F<br><br>*Experience* - I was watching my adored partner painfully disintegrate in front of my face. - P16F	What information should a person have when they become a carer for an individual with severe mental illness?<br>What psychosocial supports do carers need outside of mental health services?<br>How does becoming a mental health carer impact identity within and beyond the carer role?<br>What distinguishes mental health caring from other forms o caring for another person? What are the implications? (for the person, the carer, services, communities)<br>What are the experiences of vicarious trauma and Post Traumatic Stress Disorder among carers? - AG3<br>What further supports are required for carers (e.g. housing and Centrelink) - AG3

Message - If he had cancer, my supports would be plenty but with mental illness, we were lepers I would have liked warm referrals to counselling for emotional support. And some sense of security that he was being cared for while I was ir hospital. - P16F	What does the Carer journey look like? What if any are the stages? What impact does it have on their well-being and future? How are transitions managed? - AG3

Table S2: Categories and research questions for the theme: Care and Treatment

Experience/Message	Possible research questions
**Private vs public system**	
Experience - Private clinics are way better than the public system– P1M<br>*Message* - There shouldn't be a distinction between public and private. Public just locks you up until you are safe to be released and private needs to be more holistic. – P18F<br>*Experience* - Because of all my conditions, nobody is willing to send me to the public system. They are worried it will be too overwhelming for me. – P19F<br>*Experience* **-** APARC [Adult Prevention and Recovery Centre is a sub-acute residential care service] advised me to go to get private health cover and find a psychiatrist because my problems were too complex. – P10F<br>*Experience* - Public health (services) are very cold – P12F<br>Message - Clinicians need to have better listening skills and empathy for those in distress – P12F<br>*Message* - Better management of bulk billing services for existing patients. They shouldn't have to wait for 2 months – P11F<br>The [private clinic] was so good. The nurses were so caring and you can stay in your room for as long as you want. At the [public hospital], you had to be in the common areas most of the time and there were some violent people there… it was scary. – P21F	What makes a clinic/hospital environment more or less able to support persons with mental health problems when they are unwell?<br>What are the differences between public and private inpatient services for mental health, from the user's perspective? How can the differences be reduced?<br>What are the key improvements required for the public mental health system to provide better care?<br>What are the guidelines followed when booking appointments at bulk billing services? How can they be improved for existing service users?
**Feeling safe with healthcare providers**	

Experience - People are not going to tell their story unless they feel safe and confident. Once we get into the mental health system, finding the right person who clicks well with you is a challenge. – P4F<br>*Experience* - I was fortunate to see someone in private practice. – P5F<br>*Message* - It is important to get the right person (clinician) for yourself. You've got to feel safe. – P5F<br>*Experience* - Healthcare providers did not understand and could not identify that I had a problem and that I was not just a helicopter mum – P5F<br>*Message* - Some are stuck with a therapist for a long time. They don't open up because they don't feel safe and they don't know that they can change therapists. It's a lot about the relationship… – P5F<br>*Experience* - One psychologist was really passive. I felt like I was going to have a cup of tea with a friend. The next one was very rude. I felt she was victim blaming. – P19F<br>*Experience* - I changed 10 psychologists in 10 years because I couldn't connect to any of them – P13F	What steps can mental health services and mental health professionals take to ensure that their clients feel safe and confident to share their story?<br>How do mental health professionals distinguish between what is important and what is not, from their client's presenting problems?<br>What are the differences between the understanding of a therapeutic relationship between service users and therapists? - AG2<br>How is agency, autonomy and power navigated by people with mental health concerns and what are the consequences?<br>How does one choose the right psychologist without first having to try them out?<br>What factors promote a connection between service users and their psychologists/ mental health professionals?<br>What therapeutic practices, modalities and personal qualities of healthcare providers are the best fit for service users? - AG3<br>Why do service users need to feel a connection with their psychologists/ mental health professionals?<br>Trust – how is it built, exchanged, lost for people with mental health concerns when seeking help from health professionals?<br>What does evidence for effectiveness of therapy look like from a lived experience perspective?<br>How can choice and control be better given to the service user?
**Improving private psychological services**<br>*Experience* - I spent weeks telling the psychologist my story but no help…- P16F<br>*Message* - Psychologists need to be trauma informed. They do not necessarily understand suffering and poverty…- P16F<br>*Experience* - My therapy kept rolling over year after year for 10 years with no review of my progress or improvement. – P14F<br>*Message* - There needs to be more checks and balances with psychologists and counsellors. – P14F<br>*Experience* - Psychologists open up your wounds and leave you to come back in a week's time!!! – P9M<br>*Experience* - CBT [Cognitive Behaviour Therapy] is like being in scouts – ticking off boxes. Why would I accept to do CBT when I know that it's not going to help me? – P9M	What makes psychological therapy useful?<br>How can private psychology services be made more outcome oriented?<br>What do service users expect from their therapist?<br>What are the barriers to therapy that are experienced by psychologists? - AG3<br><br>How many sessions are needed with the Psychologist for therapy to be useful?<br>How can psychological therapy standards be implemented?<br>What supports do service users say are needed between psychology appointments? - AG2<br>Would a wider tele health service assist in creating a greater choice of services in the community? - AG1

Message - There's stuff I will not share with a woman– P9M<br>*Experience* - 5 sessions of therapy is simply not enough. My psychologist quit his job saying that he could not help his clients. – P2F<br>*Message* - We need more sessions. – P2F	
**Client choice in type of medication**	
Experience - I'm not happy with popping pills but doctors wouldn't have a bar of it – P1M<br>*Message* - Some people are not comfortable with popping pills. Accept that and find alternatives. - P1M<br>*Experience* - They wanted me to take pills. I did not want to. They threatened me with consequences – P6M	What are the barriers experienced by people with mental health problems in popping pills?<br>What alternatives exist to popping pills for people with mental health challenges?<br>What role does choice in treatments play for people with mental health challenges? How do they experience the interface with doctors when discussing medication concerns?<br>How do people self-manage and adapt in their daily lives in relation to offered medications and other treatment options?<br>What is the evidence on the effectiveness of shared decision making in medication prescriptions? - AG3<br>How often is shared decision making practiced? What are the barriers to its implementation? - AG3<br>What are the barriers to implementing social prescribing?<br>What parts of treatment do service users get a say in? Does this differ from flexibility in treatment choice allowed in service users wanting treatment for a physical illness?
**ED and mental health care**	
Experience - The assessment at ED [Emergency Department] to send me to the mental health ward was brutal. The questions they asked me and the way it was being asked was very harsh. – P10F<br>*Message* - The ED is no place for someone in a mental health crisis. – P13F<br>*Experience* - I went to an Urgent mental healthcare centre [non-governmental service] when I was acutely unwell. That is so much better than going to the ED. – P17F<br>*Message* - People in a mental health crisis need a safe space to de-escalate, be heard, get support, encouragement and validation from a peer worker. – P17F	What are the elements of care needed for persons in a mental health crisis?<br>What are the service characteristics necessary to care for an individual experiencing a mental health crisis?<br>What are the best ways of implementing the Peer-First, Peer-Last models of care? How, if ever, do they vary depending on type of mental health setting?<br>What are the non-ED models of care (e.g. Psychiatric Emergency Care centres and Safe Havens) for individuals in a mental health crisis? How effective are they?<br>How can care for persons in a mental health crisis be improved?<br>What lessons can the public system learn from the non-governmental sector on emergency mental care?
**Interactions with staff in the psychiatry ward**	

Message - People who work in mental health services really don’t care about those with mental health problems. -P7F

Experience - In the ward, I was no longer seen as an individual. I was just someone who occupied a bed that someone else was waiting for…- P16F

Experience - One told me that I laughed too much and needed to calm down but that was how I coped with the stress…- P16F

Message - Nurses make assumptions about you that can be incorrect. – P16F

Experience - I was put with people who kept having physical and emotional outbursts from drug withdrawals or psychosis. It was the worst experience of my life because I had anxiety and depression. All I needed was some time out…– P10F

Message - The mental health ward needs to be separated into segments. They can’t keep treating everyone like they don’t know what going on… - P10F

Message - It is terrible for people who have no one to advocate for them. If they do, they don’t know how to advocate to the doctors. – P5F

Message - Mental health interventions are typically short term and end before the person is confident to stand on their feet. – P4F

Message - Mental health care needs to be led by Allied health and Lived Experience personnel rather than a biomedical model with less focus on the diagnosis and more attention to their needs. – P17F

Message - Don’t blame people who are mentally unwell– P4F

Experience - They say, ‘Keep taking your tablets’. When I asked questions, they said, ‘You won’t understand unless you’re a bloke.’ – P2F

Message - Women are as clever as men – P2F

Message - Complexities are more common in people on lower income – P4F

Experience - They just don’t tell you what is wrong with you. “Oh, you won’t understand!” – P2F

Message - I need to understand what is wrong with me. – P2F

Experience - One nurse said to me, ‘No more babies. This is what it does to you.’ That was not a good thing to say to a new mum. – P21F

What do mental health service users feel about the way that mental health staff interact with them?
What do service users feel about how mental health staff should interact with them?
How, if ever, can service users who are anxious be made to feel safe in psychiatric wards?
What outcomes do mental health interventions aim to achieve in their clients?
What outcomes should mental health interventions aim to achieve in their clients?
What roles can allied health professionals play in the care of persons with mental health challenges?
How is gender-based discrimination experienced by service users when they access mental health services?
How are gendered assumptions operationalised in mental health care?
How can socioeconomically disadvantaged service users be better helped?
What factors prevent healthcare professionals from sharing health related information with service users?
How do people with lived experience define and understand the concept of health literacy?
What are the views of staff and patients regarding the culture of the ward environment (milieu)?
Why do people with mental health problems get blamed by services?
Does training of mental health staff reduce blaming?
What do people need to build confidence in long-term self-care of their mental health?
How can people with lived experience and mental health professionals effectively share power and expertise?
What are the workforce challenges faced by mental health services? - AG1

Improving inpatient care

Message – I would love to be given hints to make admission and stay easier like bringing your own pillow and doona [quilt or duvet] (a trick to at least get a good night's sleep). – P16F

Experience - I really did value the smokers circle of 'inmates' and the camaraderie developed there - possibly my first taste of an informal peer support group. I didn't smoke. Real healing started through hearing stories from individuals who were also on their journey to existential enlightenment. There was great wisdom (whatever I needed to hear to feel safe) in the 'lifers' and they were funny. Laughter was a rarity everywhere else. - P16F

Message - I'd like orientation to be carried out by one of the patients in a 'buddy system'… to tell the unspoken rules and all the inside goss (sic). Also having a 'buddy' makes walking into the full cafeteria at lunch time much less daunting. Facilities are terrifying for newbies. A good orientation is critical to being less concerned about survival in a scary place and more about getting well - perhaps a welcoming lived experience ambassador? - P16F

How can care in inpatient psychiatry wards be improved?
How can mental health wards be humanised?
What is the patient milieu in the inpatient ward?
What are the subcultures of recovery?
What are the hidden cultures of patient to patient peer support while admitted to psychiatry wards?
What do staff think patients are doing during their time in inpatient units?
How are inpatient units and social worlds navigated?
Do the boundaries required for staff at mental health wards impact the patient experience and their recovery process? - AG2
How does burnout in mental health staff impact patient recovery? - AG2
What mental health supports are available for mental health workers? - AG2
Many mental health nurses I talked to got into the field due to a mental health diagnoses in themselves or others close to them. Does the work retraumatise them? How can we support them so they can support consumers? - AG2

Trauma informed care

Experience - In ED they wanted to give me some medication anally. I flatly refused. They became angry. I said I wanted to speak to the doctor in private. When I told her why, it was all OK!! – P1M

Message - Nurses and others need to understand trauma – P1M

Message - Many therapists do not understand trauma informed care– P5F

Message - Trauma informed care is needed. Sometimes, all we need is to be heard . We do not need 'theoretical people' – P4F

Message - Healthcare providers need to be trained on what to say and what not to say to people with mental health problems. – P4F

How do service cultures and practices inform the delivery of trauma informed care?
What does mental health trauma look like in everyday exchanges with healthcare providers?
What is and is not appropriate language to use with individuals who experience mental health problems?
What training do mental health professionals receive ir 'trauma informed care'? How do they put this training into practice?
Would buddying up GPs with mental health peer workers enhance the former's understanding of menta health challenges? - AG1
What is the best way to teach doctors about trauma and trauma-informed-care for people who experience mental health challenges?
What gets in the way of healthcare providers understanding the presence of trauma and the potential impacts of their practice?
What are the elements of trauma informed care?
How can people with mental health conditions assert themselves safely within mental health systems? from the perspectives of mental health providers, consumers and family/carers? Do they align? Why or why not?
What do everyday human rights look like in the ED for people with mental health conditions?

**Need for a psychosocial advocate during a mental health crisis**	
Experience - During my episode, I made choices that shouldn't have been made. I sold my house… I couldn't think clearly. There was loss of control. I felt trapped… - P16F<br>Message - I needed a lot of help and advocacy during those times… somebody who cared… with no power imbalance… - P16F<br>Experience - Memory and Planning are skills I lack at the best of times but in psychosis I barely knew my name let alone remember what's on and when and where- P16F<br>Message - I needed reminders and check-ins and follow-ups. Verbal, written, on calendars, a timetable of activities like Yoga or art. - P16F	What psychosocial supports are needed for people during times of crisis?<br>How could individuals be better supported by allied health professionals when experiencing a mental health crisis? – AG1<br>What would advanced care plans look like from a lived experience perspective?<br>How effective are advanced care plans from a lived experience perspective? - AG3
**Peer involvement**	
Message - Bring Peer involvement to all services – ED, Psychiatrists, Psychologists, GPs… - P11F<br>*Message* - We need to have a minimum of 2 LEWs at every mental health service site. – P17F<br>*Message* - Peer workers are pivotal to mental health care and we need to listen to those with lived experience to reshape mental health services. – P18F<br>*Message* - Peer support workers can do as good a job as a psychologist to help people in distress – P9M	What if any, is the role of mental health peer work in GP and psychologist practices?<br>What roles do mental health peer workers play in mental health services?<br>How does the mental health peer work role differ in different mental health settings and contexts?<br>What are the common elements of peer support across mental health settings and contexts?<br>What qualifications or professional development would mental health peer workers benefit from, to enhance their skillset so as to ensure a duty of care is met within clinical mental health settings ? - AG1
**More integration of care**	
Message - Make psychiatrists and psychologists and social workers and counsellors and psychotherapists work together and include them in primary and secondary care. – P11F<br>Message - All the services we are using need to share notes and be linked to one another. – P12F<br>Message - People don't know where to turn and find the services they need. - P11F<br>Message - We need a case worker to bring all the services together – P11F<br>*Message* - I am able to engage openly with psychologists and psychiatrists because I am a social worker. Sadly, many counsellors and psychotherapists do not have university qualifications and are therefore not recognised or engaged by mental health professionals. - AG1	How can the different sectors of the mental health service system work together?<br>How can patient notes/records be shared between providers of mental health care, treatment and support?
**Carer acknowledgement and support**	
Experience - There was no acknowledgement from services of the trauma I went through as a carer. It was all about my son, which was fine but how do you expect the child to recover when the carer is struggling? – P3F	How must mental health services engage with carers of individuals with mental health problems?<br>Why are carers not valued and believed by mental health professionals and services?

Message - Mental health services need to care about the carer as well. Ditch the judgement and the blame. – P3F<br>Experience - I was suddenly a carer, on constant suicide watch… we'd lost his income too and were fighting Work Cover and his workplace for psychological injury (not a cheap or nice process at all). On top of doing my PhD. I was falling apart and I had no scaffolding to hoist me back up. We were worried we would lose our home - we couldn't cover mortgage payments. - P16F<br>Message - I would have loved a warm referral to mental health carers group… , some help to find emergency relief with dignity, so financial stress didn't contribute to psychosis, and help at home. A warm referral to financial planners, social worker etc.. - P16F<br>Message – Carers need to remind treatment teams of the carer's charter. - AG1	What are the psychosocial support needs of carers of persons with severe mental health challenges?<br>What barriers are carers experiencing when attempting to engage or engaging with 'Carers Gateway' and 'Carers Australia' to access psychosocial supports? - AG1<br>How can carer Strategies, Policies and Charters be realised in practice?
**Training for health professionals**	
Experience - My GP told me that he has learnt so much about mental health problems from me. – P1M<br>*Message* - There needs to be more mental health training for doctors. – P1M<br>*Message* - There is too much emphasis on doctors to manage mental health but not enough training– P5F<br>*Message* - Make GPs more aware of online programs and services such as MindSpot. – P11F<br>*Message* **-** Psychologists have no clue about complex trauma and how to look at a person as a whole. I had to teach my psychologist how to help me. – P18F<br>*Message* - Police need to be trained in mental health– P5F<br>*Message* - New International GPs move to the country and disregard mental health problems because in their culture, it is ignored – P4F	What can people with lived experience teach doctors about mental health care?<br>How do doctors include lived experience into the decisions they make about treatment and care?<br>What are GP's views of their knowledge of mental health problems?<br>What are the views and experiences of International GPs on treatment of mental disorders?<br>What are GP's attitudes towards mental health presentations, mental health care and training in mental health? - AG1<br>How effective is trauma informed care training for psychologists?<br>What, if any training, do the police receive in mental health care?
**Child protection agencies and mental health care**	
Message - Child protection do not believe you or care about what you say. They only listen to the support worker-P7F<br>*Message* - The minute you have a mental health problem as a new mother, they want to refer you to the Department of Child Protection and you have to fight to keep your baby– P4F<br>*Message* - I was alarmed when working with child protection… the lack of knowledge surrounding mental in the work force. - AG1	What, if any mental health training does child protection service staff receive? Is there a need to improve training?<br>What are the experiences of new mothers with midwifery services when they have mental health problems?<br>Would engaging mental health peer workers with child protection workers enhance outcomes? - AG1
**Hope**<br>*Experience*: This year has been transformational for me. After 40 years of struggle, I decided not to be so emotional but to starting thinking rationally. I realised that however much darkness there is, there is a light inside of us and however much trauma and problems, I have to keep facing, I find that there are more and more people around who are wanting to help and I have started engaging with them and taking a chance. Challenges will keep coming up	How do people with mental health conditions define hope?<br>How can hope be instilled in people with lived experience of severe and enduring mental health problems?<br>Would engaging with allied health professionals with lived experience enhance hope in consumers ? - AG1

from time to time but we have to keep working on it and never give up because life is precious and we must not lose hope. -P20M	

Table S3: Categories and research questions for the theme: Continuity of care

Experience/Message	Possible research questions
**Post discharge care and support**<br>*Experience* - While I was admitted in the ward, I was alright. So they discharged me. But at home there was no support and I quickly became unwell again. – P19F<br>*Message* - At the hospital, there were no stressors. No cooking, groceries or talking to people. Nurses were checking on you every hour. – P19F<br>*Message* - I think there needs to be ongoing support after you are discharged from hospital. – P15M<br>*Message* - We are discharging people back to homelessness– P8M<br>*Experience* - Once I was discharged from APARC, things deteriorated quickly again. All the pressures of 'life admin' return once you go back home. When you are admitted, all those pressures are removed. – P10F<br>*Message* - We need supports even after we are discharged from hospital and APARC. – P10F<br>*Experience* - The maternal and child nurse was only concerned about the baby and her development. She had nothing for me as a mother with postpartum psychosis. – P21F<br>*Experience* - When I got discharged and went home, there was no support. I was lucky I had my mum around. – P21F	What supports are needed for individuals who are discharged from the psychiatric hospital from the service user perspective?<br>What are care transition needs from a lived experience perspective?<br>Would gender specific care enhance outcomes post admission? – AG1<br>What supports are needed for new mothers who are discharged from the psychiatric hospital?<br>What type of care do maternal and child nurses provide for mothers with mental health problems?
**Men's mental health service needs**<br>*Message* - Where is the Men's mental health Bunnings [Hardware store]? All men love DIY [Do it yourself] but they all go to Bunnings to get the tools– P9M	What are men's mental health support needs?<br>Is mental health recovery experienced differently for men, compared with women?
**Carer needs and support**	
Experience - I had to keep taking leave to look after my son. As a mental health social worker, my employer used my excessive leave against me and I had to leave and get on a carer's pension – P3F<br>*Message* - Employers need to be trained in mental health. Every agency needs to have a dedicated carer advocate trained in mental health crisis. Just like when someone is injured at work. – P3F<br>*Experience* - I am coming to the end of my caring role but there is no support for me to get my life back. I feel unsupported and unvalued although I have spent the last 20 years looking after an acutely unwell person. I	What policies do employers have that relate to when their employees experience mental health challenges or become carers of individuals with mental health challenges? How are these policies put into practice?<br>How can carers regain an identity beyond their caring role after several years of caring?<br>How do carers develop a new identity once their caring role ends? How do they navigate this change?

have some physical health issues but my finances do not allow me to access care for them. – P3F<br>*Message* - Does anyone care? – P3F	Would more training for employers around mental health assist carers or employees transition to and from the workforce? - AG1
**Centrelink interactions**	
Experience - Getting on disability pension is a nightmare. The people at Centrelink [the agency that delivers social security payments and services to Australians] have no knowledge of the challenges we face. – P1M<br>*Message* - Centrelink treats every person like they are trying to steal presents from under the Christmas tree. – P9M<br>*Experience* **-** Centrelink sent me a notice saying I owed 1000s of dollars… -P7F<br>Centrelink asked me to do a diploma in Community services but I was not allowed to do the course like the others. -P7F<br>*Experience* - Centrelink people are typically rude, derogatory and exacting, keeping you on call for 1-3 hours at a time. I cannot do that when my son is unwell. – P3F<br>*Message* - Centrelink needs a dedicated carer team who is trained and understands mental health recovery. – P3F	How does Centrelink engage with individuals with mental health problems?<br>What should the assessment process for Disability Support Pension look like from a lived experience perspective? What would make it better for the person and family carers?<br>What, if any training do Centrelink employees receive on engaging with persons experiencing mental health challenges?<br>What is the role of a Centrelink social worker? AG1
**Housing**	
Message - We need more housing for people with mental health problems– P5F<br>*Experience* - I was transferred to 13 motels in 27 weeks for crisis accommodation. Most of them housed newly released male prisoners. It was all about funding…– P13F	What factors influence housing for persons with mental health challenges? What do people with mental health challenges say improves their housing tenure?<br>What barriers do mental health consumers experience when accessing accommodation services? - AG1
**NDIS supports**	
Message - Supports need to be for life if the disability is permanent. I should not have to advocate for my needs over and over again – P8M<br>*Message* - Giving choice and control to individuals needs to be implemented – P8M<br>*Experience* - My son is on NDIS but his support worker has no mental health training and is all at sea when it comes to managing my son's mental health challenges. – P3F<br>*Message* - NDIS needs to have mental health support workers not just disability support workers. – P3F	How does NDIS support individuals with psychosocial disability from a service user perspective?<br>What mental health training do NDIS support workers need to have?<br>What are the benefits and challenges of receiving support from non-mental health trained support workers from a service user perspective?<br>What are the roles of the NDIS support coordinator? What are the competencies they need -AG1
